# Learning from negative findings

**DOI:** 10.1186/s13584-019-0309-5

**Published:** 2019-05-01

**Authors:** Mark I. Taragin

**Affiliations:** National Insurance Institute of Israel, Jerusalem, Israel

**Keywords:** Negative studies, Negative findings, Study quality, Study design

## Abstract

A recent IJHPR article by Azulay et al. found no association between the patient activation measure (PAM) and adherence to colonoscopy after a positive fecal occult blood test result. This commentary will use that article as a jumping-off point to discuss why studies sometimes get negative results and how one should interpret such results. It will explore why the Azulay study had negative findings and describe what can be learnt from this study, despite the negative findings.

It is important to publish studies with negative findings to know which interventions do not have an effect, avoid publication bias, allow robust meta-analyses, and to encourage sub-analyses to generate new hypotheses.

To support these goals authors must submit articles with negative findings with sufficient detail to support the above aims and perform sub-analyses to identify additional relationships that merit study.

The commentary will discuss the importance of publishing articles in which the hypothesis is not proven and demonstrate how such articles should be written to maximize learning from their negative findings.

In a recent IJHPR article, Azulay et al. explored the factors associated with whether a patient underwent a recommended colonoscopy after having an abnormal result when screened with a fecal occult blood test (FOBT). They were specifically interested in whether “patient empowerment” as measured by a well-tested scale (the patient activation measure, or PAM) was associated with greater adherence to testing recommendations. Surprisingly, their study found no association (*p* value 0.774). Why did that happen? Was their study flawed? What can other researchers learn from their experience? Did they “fail” or can we learn something from this study?

In general, the publication of negative studies has been called for, primarily to overcome publication bias when performing a meta-analysis. This commentary will suggest that beyond that reason, by analyzing the various aspects of a study’s methodology, one can glean additional insights from a study with negative findings. By performing this analysis, one can also determine whether a negative study is a false negative study or a true negative study. The Azulay study will be used to illustrate these points.

The majority of researchers set forth a hypothesis and then attempt to test this hypothesis with the most rigorous study methodology possible. However, all researchers must struggle with limited resources. The “gold-standard” of a double-blind or even a triple-blind randomized study is often not feasible and frequently not even doable. Thus, researchers settle for less. The challenge a researcher faces is to find the balance between performing a meaningful study and optimizing their “investment”. One must publish or perish using limited resources. What challenges faced the designers of the Azulay study and how well did this study perform?

The authors targeted the issue of compliance with screening guidelines. This is an important health care issue which could save many lives. The ideal way to study this question would have been a prospective cohort study where patient awareness was measured at baseline, and perhaps at subsequent decision points, and then the outcome of interest, in this case a colonoscopy after screening, would be objectively measured. In addition, information would be collected on all known and suspected factors that could impact on the outcome of interest – the potential confounders.

However, a retrospective case-control study was done instead. This approach was undertaken presumably to take advantage of known screening results. Further, this method is significantly cheaper and produces results much more quickly. But, alongside these advantages, this approach introduces a number of potential problems, discussed below, some of which are discussed by the authors.

***Study population*** determines the generalizability of a study. The population is determined by what population is targeted and what exclusion criteria are applied. The study population was limited to a single health fund, which has demographic characteristics that are different from the other funds. These differences need to be described and their implications discussed. Regarding the exclusion criteria, the authors assessed medication adherence in the health fund and could have easily assessed this in the excluded population. Similarly, the authors should have described the number and characteristics of the excluded group. Without addressing these issues one cannot be certain of the generalizability of this study. On the positive side, the authors indicate that the distribution of PAM levels in their population is similar to that found in other studies.

***Selection bias*** is a potential critical problem. Who participated and who did not, and especially what caused this discrepancy, can critically affect one’s results. In this study, 54% of the target population could not be reached and 13% refused to participate. These are huge numbers and many would consider this a fatal flaw. The target for success is an 80% response rate. While the authors try to address these problems with some demographic comparisons, they could have done more. They could have compared medical factors between study participants and non-participants, including diagnoses, medications and health care utilization.

***Sample size*** is the next consideration. After we have identified our study population, do we have enough respondents to reach a meaningful conclusion? For a positive study this can be assessed by examining statistical significance. A negative study may be defined as a study showing a result that goes against the investigated hypothesis of an increased (or prevented) risk [1]. However, rejecting the investigated hypothesis (which is typically the opposite of the null hypothesis) requires a narrow confidence interval, which in turn is driven by sample size. This study had 429 participants and generated reasonable *p* values. For example, for the main question of interest, patient activation, the PAM means for the adherent and non-adherent group were 62.77 and 61.59 with a *p* value of 0.472. The *p*-value suggests that there is little difference between the groups and one would need a very large study to find a statistically significant difference. One could do a power analysis to determine how large a study would be needed to find this difference to be statistically significant. However, while statistical significance might be obtained with a larger sample size, it is unlikely that this difference would be clinically significant.

Clinical significance is a qualitative determination which is primarily driven by the importance of the outcome, the difference between the outcomes for the various alternatives, and the cost of achieving this difference. Cost includes process differences and associated side effects. For example, in this study, the outcome of importance would be preventing cancer deaths by screening. The alternatives would include the different methods to achieve better screening participation. The cost would assess the financial impact, including the ancillary results (good and bad) for each alternative method. Of note, there is a possibility of a PAM effect in the categorical analysis, where for the highest PAM score, 44.3% were in the adherent group and 39.6% were in the non-adherent group. Thus, while the analysis was not statistically significant, if one wanted to pursue this relationship it might be worth focusing on the highest PAM score group.

***Exposure and Outcome Measures*** refers to how one measures the outcome of interest and assesses potential factors that can influence this outcome. The presence of confounding (discussed below) and bias must be addressed. Patient activation, the primary exposure of interest, may be important when taking the decision to screen as well as when making the decision to follow-up on screening results. Thus, regarding PAM, the screened population may already be a select population, a form of bias. Furthermore, because of the retrospective assessment of PAM, patient activation might have been influenced by the test results themselves, another form of bias.

The authors acknowledge that “patient activation may vary with time and context” yet do not provide us with any literature describing the stability of this measure over time. For patient activation to be useful one must know if it is stable over time and if it can be modified.

Even if PAM cannot be modified, it could be used to optimize different strategies for targeting different populations. The authors’ primary hypothesis is that there is an association between patient activation and the decision to follow-through on a screening result. However, the importance of patient activation may vary with disease, screening approaches, and interventions. A perusal of the PAM website [2] reveals that a number of studies did not find an association between patient activation and the outcome being studied. The authors should have described this and whether any similar factors were present in their study. Their study could contribute to the PAM literature by exploring in which populations PAM is significant and why.

***Potential confounders*** must be properly assessed. The authors provide a review of what factors are associated with non-adherence, including many that they did not assess, e.g., health status, patient knowledge, fear of undergoing CRC (colo-rectal cancer) screening, high self-efficacy, risk perception, and perception of the chance of developing CRC. Furthermore, the authors identified a local study which demonstrated that higher educational attainment and higher self-efficacy were important factors associated with non-adherence. However, despite doing a phone interview, the authors did not report results on any of these known factors. One can surmise that either they did not evaluate these factors, or that they plan an additional paper with those results. Nevertheless, in Table 1, the “Characteristics of study population by colonoscopy adherence”, the authors present findings on additional potential confounders. Although no differences were statistically significant, this could be a sample size issue. For example, country of birth, ethnicity, BMI, and smoking with *p* values of 0.15, 0.264, 0.118, and 0.066 respectively, may have reached significance with a greater sample size. This is especially important when planning further studies.

***Statistical methods*** are generally not a problematic issue, especially for articles published in serious peer-reviewed journals. Yet, it is worth noting that there are statistical methods to deal with negative results. The basic ones, described above, are *p* values and confidence intervals. In addition, there are methods to quickly estimate a maximum effect, such as described in the paper “If nothing goes wrong is everything all right?” [3]. In that paper the authors describe a rule of thumb “3/N” where N is the sample size where no effect was found. Thus, for example, if 20 patients were reported to have no outcome, then the upper confidence interval can be estimated as being 3/20, 15%. In general, despite our attraction to numbers, typically, study quality is much more important.

All of the parameters discussed above will determine the quality of the study. In recent years, the importance of study quality has been increasingly recognized. For example, many meta-analysis papers perform sub-analyses that evaluate the effect of study quality on conclusions. These papers often find large differences in results when stratifying by quality. Primarily driven by the need to do meta-analysis, an alphabet of tools has been developed to evaluate the quality of studies, e.g., AMSTAR, PRISMA, and STROBE [4–7]. While these tools are ultimately subjective, their structured format ensures a more complete and transparent process that can be reproduced by others – and allows evaluations to be compared. The need for, and the development of, these tools stresses the fact that there is a wide spectrum of quality among studies. This variance in study quality can partially explain why different studies of the same issue get different results and why some studies have significant findings and others don’t.

The public as well as physicians are frustrated by scientific “flip-flops” with changing recommendations over the years, e.g., hormonal therapy for post-menopausal women, cut-offs for treating hypertension in the elderly, and PSA screening, to name a few. The evaluation of study quality and the publication of negative results have the potential to generate more transparent results and better explain the variation between study results. Not only will this enable researchers to reach a better understanding of what they are studying, but this will also allow more robust models of what factors drive specific outcomes. Understanding the impact of study quality on results should also facilitate the scientific community’s ability to explain conflicting results to the public and regain the trust the public has lost in the scientific literature [8].

The quality of a study should be evident, in part, in the completeness of the discussion section. The authors of the Azulay paper did a very nice job of evaluating their results and comparing them to other relevant studies in the field of screening and PAM. In general, authors have the best knowledge of the strengths and limitations of their study. A thorough discussion not only allows better understanding of the value of a study and what future work needs to be done, but it also reflects on the knowledge and skill set of the authors. Sharing and discussing study flaws and study limitations displays the authors’ knowledge of the field they are studying and their understanding of study methodology. The discussion section should describes how reality differed from what was planned and generates the approaches needed to further develop this area of study. As noted above, the ideal prospective study is expensive and time-intensive. Lower quality studies form the basis for creating better future studies and are appropriate when beginning to study a new area.

Study results can be categorized into “positive” and “negative” but really should be more often labeled “mixed” or “I don’t know”. As described above, study quality can render a positive study’s results fatally flawed and misleading. Alternatively, despite sample size issues, a negative study can be informative. Indeed, the examples noted above illustrate how the Azulay study contributes to better understanding of potential confounders and PAM. Thus, labeling a study as negative is deceptive and should be avoided.

The need for multiple studies to form a basis for understanding is clear. Conflicting results should not be surprising and should form the basis for a more comprehensive understanding of the area being investigated. Researchers need to discuss the limitations of their studies and be more willing to admit that their results are unclear. Overconfidence is dangerous [9, 10]**.** As I learned in medical school, the best physicians know when to say “I don’t know” and are not afraid to say so. Only thus can “truth” be sought, and hopefully, revealed.

## Conclusions

Azulay and colleagues targeted an important health care decision with a reasonable hypothesis. As is the case with most studies, their study design and methodology had several flaws. Despite these flaws and the lack of a finding of an association, much can be learned from the Azulay study. It contributes to the knowledge of the importance of patient activation and may help us better understand when patient activation plays an important role. If Azulay and colleagues still believe that patient activation is associated with CRC/FOBT they will need to invest more resources to assess this potential relationship, and better assess the relevant confounders.

This commentary has used the Azulay paper to demonstrate the importance of publishing studies with negative findings. Perhaps, in this clinical setting, patient activation is an intervention which does not have an effect. Publishing this result can help others avoid investing in this intervention and encourage looking for better alternative interventions. By publishing a negative finding on patient activation other authors can use these results in a meta-analysis on the effectiveness of patient activation. Finally, as discussed above, some of the sub-analyses in the Azulay paper suggest additional hypotheses that can be pursued.

I hope that this commentary on the Azulay study will encourage the IJHPR and other journals to publish negative studies more often. Perhaps journals should also publish a statistic of how many negative studies they publish. This statistic would enable the creation of a benchmark for what percentage of studies are expected to be negative, encourage the publication of negative studies, and help identify which journals are promoting better quality research results by diminishing publication bias.

## Abbreviations

CRC: Colo-rectal cancer
FOBT: Fecal occult blood test
PAM: Patient activation measure
PSA: Prostate specific antigen
